# Predicting anatomic therapeutic chemical classification codes using tiered learning

**DOI:** 10.1186/s12859-017-1660-6

**Published:** 2017-06-07

**Authors:** Thomas Olson, Rahul Singh

**Affiliations:** 1 Department of Computer Science, San Francisco State University, San Francisco, CA USA; 2 Center for Discovery and Innovation in Parasitic Diseases, University of California, San Diego, CA USA

## Abstract

**Background:**

The low success rate and high cost of drug discovery requires the development of new paradigms to identify molecules of therapeutic value. The Anatomical Therapeutic Chemical (ATC) Code System is a World Health Organization (WHO) proposed classification that assigns multi-level codes to compounds based on their therapeutic, pharmacological and chemical characteristics as well as the in-vivo sites(s) of activity. The ability to predict ATC codes of compounds can assist in creation of high-quality chemical libraries for drug screening and in applications such as drug repositioning. We propose a machine learning architecture called tiered learning for prediction of ATC codes that relies on the prediction results of the higher levels of the ATC code to simplify the predictions of the lower levels.

**Results:**

The proposed approach was validated using a number of compounds in both cross-validation and test setting. The validation experiments compared chemical descriptors, initialization methods and classification algorithms. The prediction accuracy obtained with tiered learning was found to be either comparable or better than that of established methods. Additionally, the experiments demonstrated the generalizability of the tiered learning architecture, in that its use was found to improve prediction rates for a majority of machine learning algorithms when compared to their stand-alone application.

**Conclusion:**

The basis of our approach lies in the observation that anatomical-therapeutic biological activity of certain types typically precludes activities of many other types. Thus, there exists a characteristic distribution of the ATC codes, which can be leveraged to limit the search-space of possible codes that can be ascribed at a particular level once the codes at the preceding levels are known. Tiered learning utilizes this observation to constrain the learning space for ATC codes at a particular level based on the ATC code at higher levels. This simplifies the prediction and allows for improved accuracy.

## Background

Discovery of efficacious drugs against diseases is one of the key challenges of modern science. Drug discovery efforts typically start by screening a large number of compounds to identify “leads” which subsequently undergo optimization and in vivo test of efficacy and pharmacokinetics to identify candidates for clinical trials. The selection of a large number of compounds for primary screening is often driven both by the need to capture chemical diversity, and also because small structural variations can cardinally influence binding against a target. However, outside general principles such as the Lipinski rules [1], few rigorous criteria exist to guide selection of the initial set of molecules for primary screening. Finally, repositioning an existing drug to a novel pathology is an alluring, though limited, alternative to de novo drug design [2]. In both the above problems formulations, the ability to identify compounds that are therapeutically of interest vis-à-vis a particular pathology is critical.

Automatically determining the ATC code of a compound constitutes an attractive approach to both these problems. Towards this goal, we present a learning architecture called tiered learning that can be utilized by any prediction method (classifier) to obtain highly accurate ATC predictions. The proposed architecture is based on the premise that while chemical compounds may exhibit polypharmacology, that is, compounds may modulate multiple targets, this phenomenon has limits and anatomical-therapeutic biological activity of certain types must preclude activities of many other types. In other words, ATC classes must have characteristic distributions, which become increasingly specific as one traverses the ATC classification levels.

**Fig. 1:**
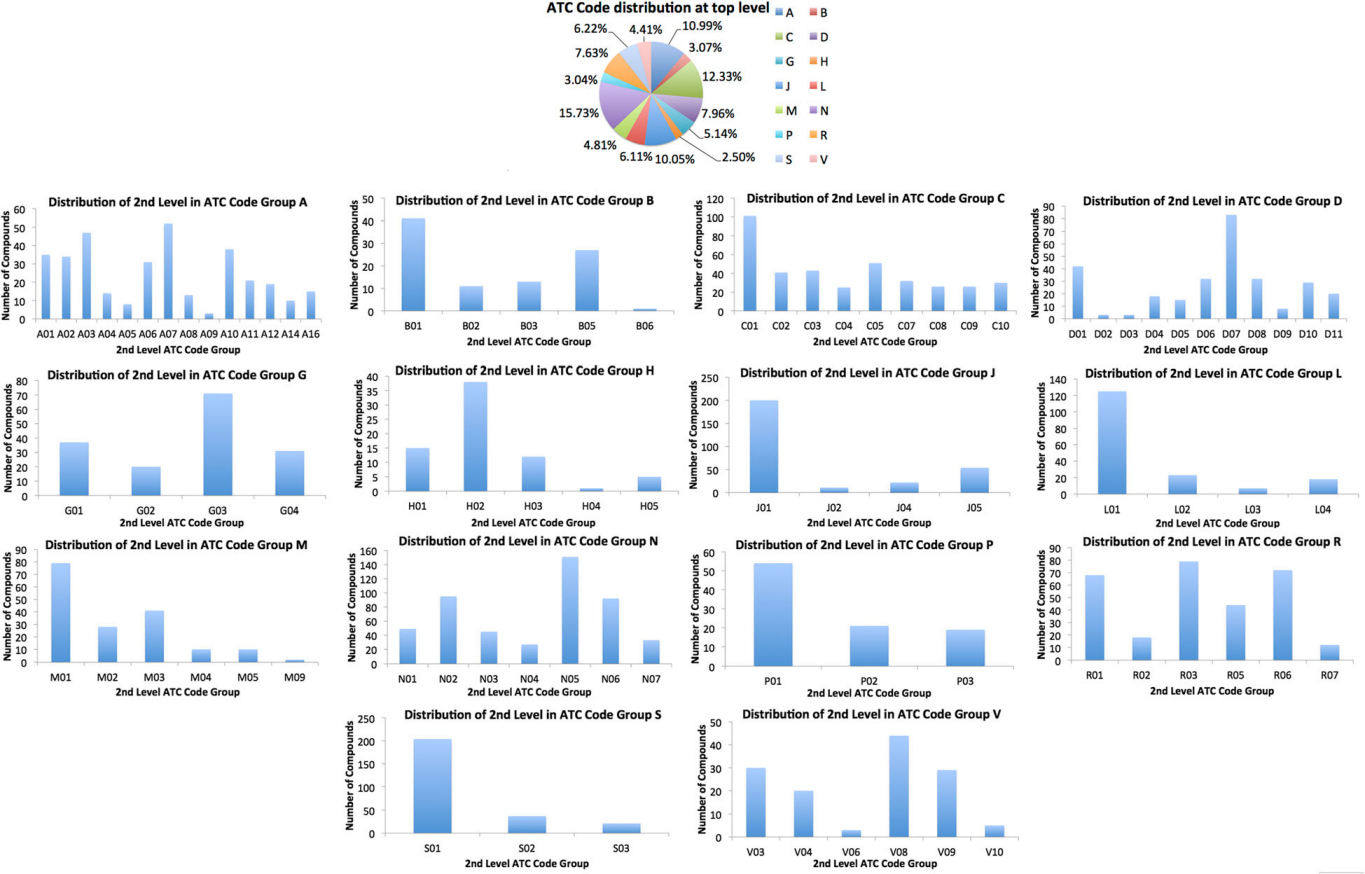
The empirical distribution of ATC codes at the top level (Pie chart) and at the second level for each of the fourteen anatomical groups in a dataset of 2232 compounds from the ChEMBL used in this study. The pie chart presents the distribution of ATC codes at the anatomical group-level. The bar charts present the distribution of compounds within each of the anatomical groups. It is apparent that each anatomical group has a characteristic distribution, which becomes increasingly specific as one traverses the ATC hierarchy

To motivate this premise, in Fig. 1, we plot the distribution of ATC classes for the data used by us at the first and second code levels. As can be seen from this figure, for each ATC class at the first level, the distribution of second level is highly specific. Figure 1 thus underlines the existence of characteristic distribution for ATC codes–an observation that can be employed to constrain the search-space of possible codes once preceding levels are known. Based on this premise, in tiered learning, prediction of the ATC code at a certain level is constrained by the ATC code at the higher levels. An advantage of such an approach is that with each successive prediction, the learning space not only becomes smaller but also more specific, reducing thereby the informational heterogeneity and simplifying the learning task. In parallel, the biochemical data used to train the prediction methods also becomes more specific in terms of its therapeutic/pharmacological content.

### Anatomical Therapeutic Chemical codes

Created by the World Health Organization (WHO) to aid in accurately performing drug consumptions studies, the ATC system assigns a code to drugs based on their therapeutic and pharmacological properties. The ATC system has a tree-based hierarchy with five levels, each describing a new level of detail of a drug’s therapeutic profile as described in the following: *first level*: One letter signifying which of the 14 anatomical groups the drug acts on. *Second level*: two digits that represent the therapeutic group of the drug. *Third and Fourth levels*: one letter each specifying therapeutic and pharmacological subgroups. *Fifth level*: two digits that are used to identify the drug within its group. Consider, for example, the drug Aspirin, or acetylsalicylic acid, which is known to have fever and pain relieving properties. Table 1 lists the ATC Codes for Aspirin. As can be seen, the ATC codes represent each therapeutic effect of the drug and the information becomes increasingly specific as we go down the code hierarchy.

**Table 1:** ATC Codes (in bold) of aspirin

ATC Level	ATC Codes of Aspirin	
	*Antiplatelet effects*	*Anti-fever/pain reliever*
1: Anatomical main group	***B*** : Blood and Blood forming agents	***N*** : Nervous System
2: Threaputic main group	**B** ***01*** : Antithrombotic Agents	**N** ***02***: Analgesic
3: Therapeutic/pharmacological subgroup	**B01** ***A*** : Antithrombotic Agents	**N02** ***B***: Other analgesics and Antipyretics (fever reducers)
4: Chemical/therapeutic/pharmacological subgroup	**B01A** ***C*** : Platelet aggregation inhibitors excl. heparin	**N02B** ***A***: Salicylic acid and derivatives
5: Chemical substance identifiers	**B01AC** ***06*** : Acetylsalicylic acid	**N02BA** ***01***: Acetylsalicylic acid

### Prior work

The problem of ATC code prediction has received significant recent attention. In [3], a two-step approach was used to predict the ATC code of a compound. In the first step, chemical-chemical interaction data from the STITCH database [4] were used based on the hypothesis that drugs that have a high STITCH interaction confidence score would share the first letter of their ATC code. For compounds which lack a STITCH interaction confidence score, the second step of the method was invoked. In this step, code assignments were made based on chemical similarity. That is, the ATC code of a compound was assigned to that of its chemically most similar counterpart. A prediction accuracy of 73% was reported in [3]. Since compounds often have more than one ATC code, in [3] the top two predicted ATC codes for each compound were used for calculating prediction accuracy; essentially, each compound-ATC code pair was treated as a unique entity. An updated version of this method was published in [5]. In it, drug ontology terms from ChemEBI Ontology [6] were employed as an additional feature to improve prediction accuracy. Specifically, the number of gene ontology terms shared by an input compound with a database compound (for which the ATC code is known) was used, since a large number of such shared terms may imply similar anatomic-therapeutic action. A prediction accuracy of 75.9% was obtained with this updated method.

Another state-of-the-art method is SuperPred [7]. The original method predicted ATC codes using 2D chemical structure similarity and reported an accuracy of 67.6% at code depth of five. An updated version of SuperPred [8] employed 2D structure similarity, fragment-based similarity, and 3D structure similarity. 2D structure similarity was defined as the Tanimoto similarity of Extended Connectivity Fingerprints (ECFP) [9]. For determining the fragment similarity of two compounds, the corresponding *n* and *m* fragments were obtained using the linker rule [10]. Next, the fragments were used to create a matrix of size *n* × *m*, which contained the Tanimoto similarity of all fragment-pairs. The similarity scores of the ^*n*^ *C*_*m*_ fragment combinations were next obtained from this matrix and used for determining the most similar fragment correspondences. Finally, 3D similarity was determined by structural superimposition computed over 100 low-energy conformers of each compound. The prediction accuracy of SuperPred was reported to be 75.3% at the fifth ATC code level. When the method was used to predict the top level ATC code only, an accuracy of 80.3% was achieved. It should be noted that the SuperPred data sets consisted of compounds that were structurally similar for each ATC group and compounds with multiple ATC groups were excluded. Additionally, certain (similar) ATC codes were combined into single groups [7]. For example corticosteroids, moderately potent’ (ATC: D07AB), ‘corticosteroids, potent’ (ATC: D07AC), ‘corticosteroids, very potent’ (ATC: D07AD) and ‘corticosteroids, plain’ (ATC: S01BA) were all combined into a single coricosteriod class. These steps helped simplify the learning task.

A Support Vector Machine (SVM) was used in the NetPredATC method [11], which reported prediction accuracies ranging between 74 and 76.5% on four different sets of compounds. The kernel of the SVM for a drug pair-ATC code pair involved information relating both drugs and ATC codes. In particular, information on the structural similarity between the drug pair was calculated using Simcomp [12], a graph based structural similarity method, or the sequence similarity between the targets of the drugs as calculated by the Smith-Waterman Algorithm [13] was used. The distance between the pair of ATC codes in the ATC hierarchy was also included in the kernel. Experiments showed that NetPredATC achieved higher accuracy when using the target similarity over the chemical similarity.

In [14], information related to compound structure, chemical-chemical associations, gene expression, target information and similarity of side-effects is utilized to predict ATC codes. Features were ranked both in terms of their importance to classification as well as their redundancy using the minimum redundancy maximum relevance feature selection method [15]. Logistic regression was used for evidence combination and prediction. In particular, chemical similarity was assessed by the Tanimoto coefficient using FP2 fingerprints [16], which are hashed based fingerprints created from all the possible fragments of molecules up to 7 atoms in length, and functional group vectors, which are binary vectors that indicate presence or absence of functional groups. Chemical-chemical associations information was obtained from the STITCH database [4], side effect profiles were obtained from the SIDER database [17], and gene expression profiles were retrieved from the Connectivity Map [18]. Target profiles were represented by a vector where each element represented a particular protein and the presence of a “1” or “0” in that position indicated whether or not the compound targeted that protein. The results reported in [14] show that this approach results in improved ROC curves as compared to other methods.

Of other methods, in [19], the hypothesis that drugs interacting with the same domain tend to share therapeutic effects was utilized to predict ATC codes. In [20], a Bayesian approach was employed to predict ATC codes for compounds restricted to the C (Cardiovascular) group. In this method, a vector of Medline terms [21] was used to represent each compound and ATC codes were inferred using the similarity of the term-vectors. The method reported an overall accuracy of 77.12% on a test set of 114 cardiovascular drugs using a training set of 390 other cardiovascular drugs. A summary of the key ATC code prediction methods in terms of data sets, descriptors, prediction algorithm, and accuracy is provided in Table 2.

**Table 2:** Summary of key ATC prediction methods from the literature

Method	Method details					
	Data Set Size (compounds)	Data Source(s)	Descriptors	Prediction Algorithm	Accuracy at ATC Depth 1	Maximum Accuracy and Prediction Depth
SuperPred [8]	2650 (for drug classification)	Transformer database, SuperTarget, ChEMBL, and BindingDB	2D, fragment, and 3D Structure-based	Consensus-based	80.90%	75.1% at a depth of 5
Chen et al. [5]	3934	KEGG	Chemical interactions, structure and ontology	Hybrid Method	75.70% (internal validation set)	75.70% (internal validation set)
Wang et al. [11]	790	KEGG BRITE, DrugBank	Information from chemical structures, target proteins, and ATC Codes.	Kernel method and SVM classification	74%	74% at depth 5.
Gurulingappa et al. [20]	504 (training + test)	Medline	Concepts generated from Medline terms	Naïve Bayes	77.12%	77.12% at depth 4

Note that methods may define the notion of prediction accuracy differently. Consequently, any comparison of numeric accuracy values should factor in the definitions

The research described in this paper varies cardinally from these prior works in its philosophy; our emphasis here is neither on feature design nor on design of new predictors. Rather, we employ the fact that each ATC code hierarchy has a characteristic distribution to design a tiered learning architecture, where the prediction of the ATC code at a particular level is constrained by the ATC code at higher-levels. This learning architecture is generic and can therefore be used to improve the performance of most prediction methods and/or feature design strategies.

## Method

### The tiered learning architecture

Like the other works surveyed in the previous section, we too employ a supervised formulation to solve the ATC code prediction problem. The novelty of our idea for predicting the ATC code of a compound lies in the proposed tiered architecture for learning and prediction. The use of a tiered learning (TL) architecture is based on the observation, as underlined by the data in Fig. 1, that anatomical-therapeutic biological activity of certain types must preclude activities of many other types. That is, successive levels of the ATC hierarchy map to increasingly conserved and distinct parts of the chemical space. In TL, during the training phase at the *k* ^th^ level, the ATC codes of the previous (higher) *k*-1, *k*-2, …, 1^st^ levels are taken into account to train a predictor. In the testing phase correspondingly, ATC codes at each level are predicted by selecting an appropriate predictor instance based on the prior (predicted) code values. Determining the code at the 1^st^ level (or root of the ATC classification hierarchy) constitutes the initial value determination problem, for which a number of strategies are proposed *vide infra*.

TL hierarchically tessellates the ATC learning space based on the premise that the distribution of ATC codes becomes progressively specific as we traverse the ATC hierarchy. In it, specific classifier instances are created for predicting the code at the *k* ^th^ level based on the prior codes. Operationally, for a compound, after the first letter of ATC code is determined by solving the initial value determination problem, the training data is filtered so that only compounds sharing the predicted ATC code of the prior level are used as the training set for the subsequent level. This new filtered training set is then used to train a new classifier instance in order to predict the next level. In this manner, a new classifier is trained for each *k*_th_ level as predicated by the codes in the previous levels.

**Fig. 2:**
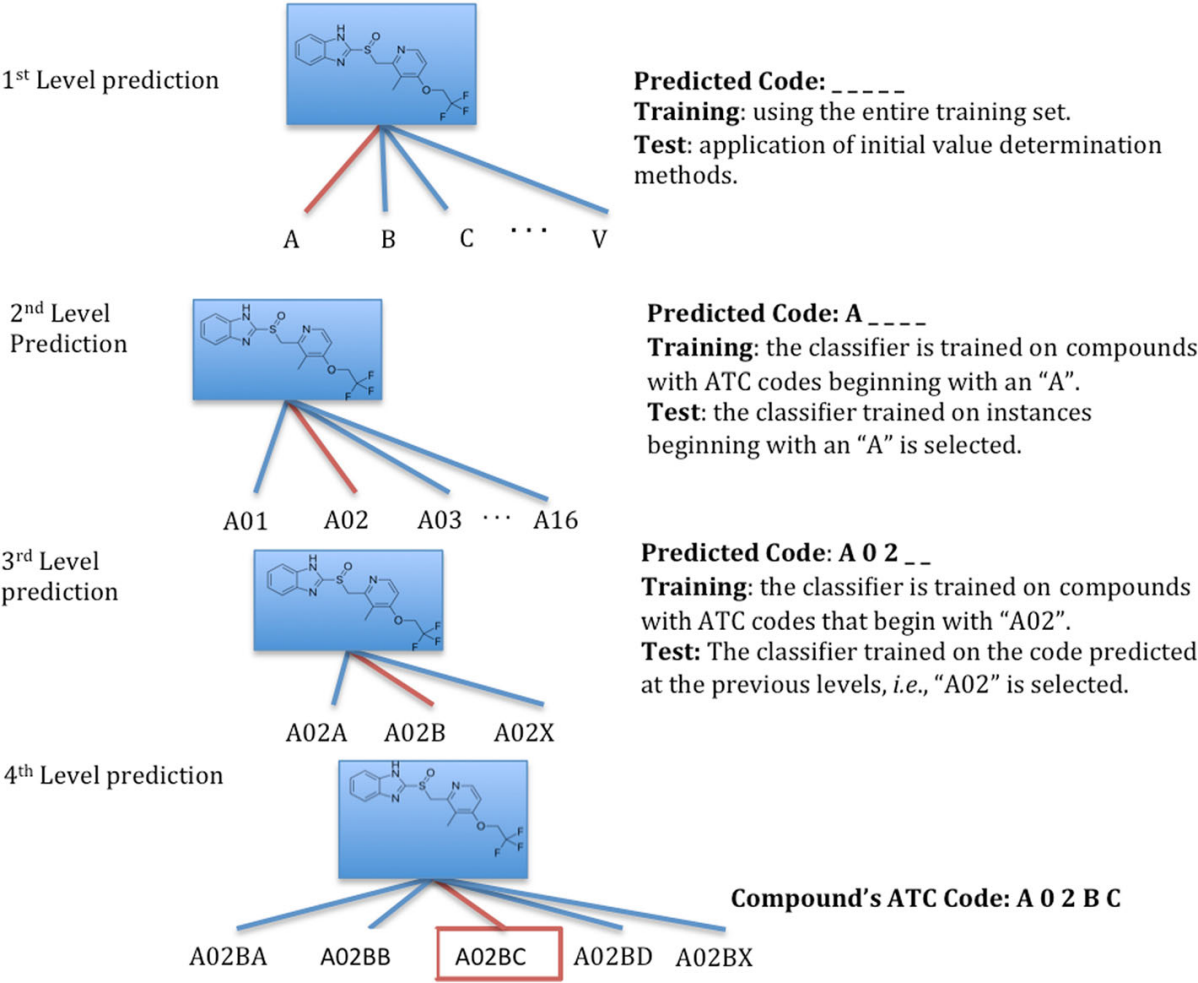
Training and prediction using tiered learning. This figure illustrates the TL architecture for a compound with the ATC code “A02BC”. At each level of the prediction, the prior predicted code is used to successively refine the learning task by creating (during the learning phase) or selecting (during the test phase) a new classifier instance

An illustration of this process is presented in Fig. 2. It presents the case where the ATC code for the first level of a compound is predicted to be an “A”. The second level is then predicted using a classifier instance trained using only such compounds which have an “A” as their first level ATC code. Continuing the example, if the predicted ATC code at the 2^nd^ level is “02”, then the next classifier instance is trained using compounds that start with the ATC codes “A02” for the first two levels. This process is repeated for each subsequent level, leading to an ensemble of increasingly focused classifiers that progressively tessellate the ATC space. For predicting the complete ATC code of a compound, a total of four levels of classifier instances would need to be trained. Since the fifth level ATC code is a chemical substance identifier, in this paper, we limit ourselves to predicting the first four levels.

Finally, a number of variations in applying the tiered learning architecture are possible. For instance, chemical similarity can be used to tessellate the chemical space, with each tessellate containing similar compounds. Subsequently, TL can be applied within each tessellate to predict the ATC code.

### Strategies for initial value determination

Determining the ATC code at the first level seeds the TL process. Since there are no prior codes to constrain the choice, this constitutes and initial value determination problem. We propose the following two strategies to determine the initial code value for seeding the TL process. Their comparative assessment is conducted in the experimental section.

- *Single-level prediction using supervised learning*: In the approach, the initial value is determined through supervised classification involving the entire training data. That is, a special classifier is trained to predict the first level ATC code only.
- *Seeding using external knowledge*: In this approach, the initial code value is determined using external knowledge, including but not limited to seeding using another predictor, use of prior chemical knowledge, use of ATC information in repositories like ChEMBL, and use of information from literature.

### Classifiers used as part of tiered learning

In this work, four learning algorithms (implemented in the Scikit-learn python library [22]) are used as in the TL architecture. These include: Naïve Bayes, Multilayer Perceptrons (MLP), Support Vector Machine (SVM), and the Random Forest. These algorithms represent a diverse set of learning philosophies to the machine learning problem.

### Descriptor design

In order to construct the descriptors used in our work, we extracted structural and interaction data from the STITCH [4] database. STITCH is a repository for compound interaction data and contains data on 300,000 small molecules and 2.6 million proteins from 1133 organisms. The database includes information on associations between chemicals as well as associations between chemicals and targets. A numeric score represents the strength of these associations. We use structural information from the ChEMBL database [23] as well as the aforementioned information from STITCH to design three types of chemical descriptors (feature vectors) that are described in the following.

### Representation of compounds based on chemical structure

A molecule can be represented at various levels of abstraction, starting from simple abstractions such as the chemical formula to highly complex ones such as surface-based representations [24] and ultimately the Schrödinger equation. In our work each molecule is structurally represented by its chemical fingerprint. A fingerprint is a linear bit-string representation of the connectivity of a molecule. Advanced fingerprint algorithms can also incorporate atom types and other physical-chemical properties. In particular, two types of chemical fingerprints are used by us: the RDK Linear Fingerprint and the RDK Morgan fingerprints [25]. The RDK chemical fingerprint is a linear fingerprint with each element in the bit string indicating the presence or absence of a particular structural motif. The fingerprint length is a parameter and can be set to encode different numbers of features. Further, the fingerprint path-size is another important parameter which dictates the maximum size in bond lengths of the encoded features each bit represents. For our method, we use a fingerprints length of 2048 and a path-size of seven.

Morgan fingerprints differ from the RDK linear fingerprint in that they encode neighborhoods or groups of adjacent atoms in the molecule, as opposed to simply encoding the presence or absence of specific atoms and substructures. Morgan fingerprints are created through an iterative process. Initially, each atom in the molecule is represented by an integer value. Next, these integers are combined iteratively based on proximity of the corresponding atoms. Integer values corresponding to atoms that are separated by a single bond are combined in an array and a hash function is applied. This process iterates till a preset inter-atomic radius (set to four in our case) is reached. Finally, the integers are stored as a binary sequence. Both fingerprint-based representations allow for rapid 2D structure representation. In the following, we abbreviate the chemical fingerprint-based descriptor as CFP.

### Representation of associations between compounds

**Fig. 3:**
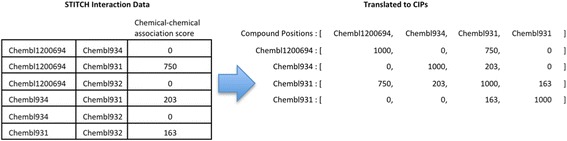
Creation of chemical interaction profiles (CIP) from STITCH data. The CIP is encoded as a vector, where each position represents the chemical-chemical interaction of the compound to another compound in the database. These vectors are created using chemical-chemical association data from the STITCH database. Compound-target interaction profiles, which use compound-target interaction data are constructed analogously

Here, we seek to design a descriptor that allows capturing the context of a compound based on its associations with other compounds. To do so, we utilize chemical-chemical association data from the STITCH database [26]. In STITCH, associations between chemicals are established using structural similarity, reactions from pathway databases, literature-based associations, and similarity of activity based on Medical Subject Headings (MeSH) pharmacological action terms and activities in the NCI60 cell lines. The combined chemical-chemical association score between two compounds in STITCH ranges between zero and 1000, with the score of zero corresponding to no discernable association and the score of 1000 implying high association. To utilize these chemical-chemical association scores, we create what we term hereafter as the chemical interaction profile (CIP) for each compound. A compound’s CIP represents its similarity to other compounds in the database and is based on the corresponding combined STITCH association scores. A CIP is encoded as a vector, where each element represents the association of the given compound to another specific compound in the database. Associations that are unknown or absent are encoded by a zero. Compounds can be compared by computing various similarity measures between their CIP representation vectors in a manner similar to that for chemical fingerprints. The process of constructing a CIP is illustrated in Fig. 3.

### Representation of compounds based on interactions with similar targets

This descriptor is motivated by the observation that for compounds a large number of interactions with the same proteins or enzymes (targets) putatively indicate shared therapeutic activity. The chemical-target interaction data are extracted from the STITCH database and include interactions such as bindings, inhibitions, and other interactions. We only use high-confidence interactions, represented by the combined STITCH interaction confidence scores equaling or exceeding 700 and limit ourselves to only interactions occurring with human proteins. The chemical-target interaction profile abbreviated hereafter as CTP is created analogously to the CIP; for a given compound, each element of its CTP vector represents similarity with another compound in the database. The similarity score is defined as the number of shared (common) targets between the two respective compounds based on the data extracted from STITCH.

## Results and discussion

### Training and test data

The data used for training and testing the proposed learning architecture consisted of 2232 molecules selected from ChEMBL [23]. The ChEMBL database is a manually curated repository of compounds and currently contains over 1 million compounds. However, only a small set of these compounds have associated ATC codes. We selected compounds that were either approved drugs or had reached phase-III trials and had high quality information associated with them including information on their ATC code, targets, and method of action (MOA). Of the 2232 compounds, 1674 constituted the training set and 558 made up the test set. Each compound was represented using the CFP, CIP, and CTP descriptors.

### Assessment of descriptors in terms of prediction accuracy

**Fig. 4:**
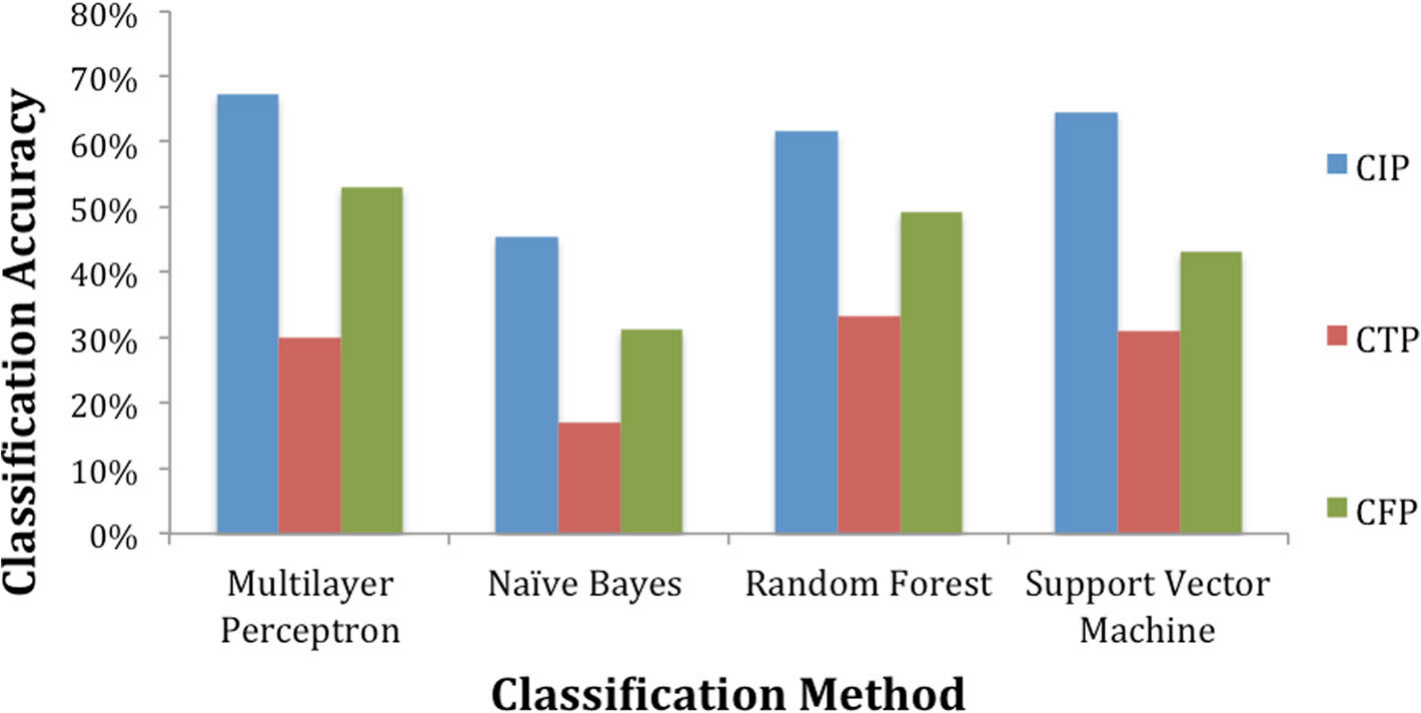
Prediction accuracy of each descriptor at the top-level of the ATC code. The CIP descriptor led to the highest prediction accuracies

The aim of this experiment was to assess the three descriptors used by us, namely, CFP, CIP, and CTP in terms of their importance for prediction accuracy. Towards this, each descriptor was used in turn to train the prediction methods on the 1674 compounds in the training set. Next, each descriptor-predictor pair was used to predict the first through fourth levels of the ATC hierarchy for the 558 compounds in the test set without TL. Henceforth, we shall call this setup as the standard training and prediction setting and abbreviate it as the STP setting. The classification accuracy defined as the percentage of the test set for which the ATC codes were correctly predicted for each of the descriptors at the first level is graphed in Fig. 4. In terms of the individual descriptors, the CIP had the highest overall accuracy of 72.40% and was also the most accurate descriptor across all classifiers. CFP was the second most accurate descriptor, followed by CTP. The performance of the classifiers at predicting each level of the ATC code for CIP and CFP is presented in Table 3.

**Table 3:** Accuracy of classifiers at levels one through four of the ATC code in the STP setting

STP prediction accuracy at each level of the ATC code								
ATC Level	1		2		3		4	
Descriptor	CIP	CFP	CIP	CFP	CIP	CFP	CIP	CFP
MLP	**73.66%**	**53.05%**	**67.56%**	**43.37%**	**59.14%**	41.22%	33.87%	34.05%
Naive Bayes	47.67%	31.18%	36.38%	17.03%	30.47%	17.20%	22.76%	12.19%
Random Forest	66.49%	49.28%	58.96%	40.32%	50.18%	37.46%	28.49%	32.26%
SVM	72.22%	43.19%	64.52%	42.29%	58.60%	**43.19%**	**41.22%**	**38.35%**

The classifiers were trained on 1674 compounds in the training set and tested on the 558 compounds using the CIP and CFP descriptors. The highest prediction accuracy at each ATC code level is highlighted in bold

To further assess the CIP and CFP descriptors, five-fold cross-validated predictions were separately performed using the combined training and test datasets. The results are summarized in Table 4. Here too the CIP descriptor was found to outperform the CFP descriptor. We note that the classifiers trained on the entire data in this experiment were solely constructed to evaluate the CIP and CTP descriptors and were not included in other experiments. Based on these results, the CIP was used as the primary descriptor for experiments going forward, as it encompasses both chemical association information and compound structure.

**Table 4:** Five-fold cross-validated prediction accuracies for the CIP and CFP descriptors on entire dataset (2232 compounds)

Fivefold cross validation results across entire dataset								
ATC Level	1		2		3		4	
Descriptor	CIP	CFP	CIP	CFP	CIP	CFP	CIP	CFP
MLP	**65.82%**	32.38%	**58.59%**	31.06%	**53.02%**	27.79%	**39.58%**	24.07%
Naive Bayes	46.48%	22.51%	32.23%	17.41%	30.13%	14.81%	24.30%	8.91%
Random Forest	60.08%	37.51%	53.45%	28.77%	47.01%	25.28%	36.11%	22.68%
SVM	64.69%	**39.04%**	56.53%	**31.92%**	52.27%	**28.69%**	39.28%	**25.60%**

At each level, the highest prediction accuracy is highlighted in bold

### Baseline performance of the prediction algorithms

Table 3 quantifies the baseline performance of the four prediction algorithms on our data set. These results show that the MLP and SVM methods had the highest prediction accuracies depending on the level of the ATC code being predicted and the descriptor used. MLP outperformed all other classifiers at the first through third level when using CIPs with an accuracy of 73.66, 67.56, and 59.14%, but then yielded to the SVM at the fourth level, which had an accuracy of 41.22%. For CFP, the highest prediction accuracy was obtained with MLP for the first and second levels of the ATC code. However, at the latter two levels, SVM performed better than the rest of the classifiers. From these results, in terms of prediction accuracy in the STP setting, MLP emerged as the overall best ATC predictor, with SVM as the second best.

### Experimental analysis of tiered learning

To experimentally compare the proposed TL approach with STP, we trained and tested each of the four prediction methods by incorporating them in the TL architecture and compared the prediction accuracies with those obtained with the same prediction methods in STP settings. We note that application of TL requires solving the initial value determination problem. We assessed two strategies for initializing TL, namely single-level prediction using supervised learning, and seeding using external knowledge. For the latter strategy, we seeded the TL process both by using SuperPred and by using information from ChEMBL. The following three measures were used to evaluate the prediction performance of TL:

- *Prediction accuracy at level k:* Reflects the accuracy in predicting the ATC code at a level *k* and is computed independent of the prediction accuracy at other levels. Hereafter, this measure is denoted as *A*(*k*). In principle, the index *k* varies from level 1 to level 5. However, depending on how the initial value computation was done, this measure may be trivial for level 1.
- *Cumulative prediction accuracy till level k*: Reflects the average accuracy of prediction starting from level 1 till level *k* inclusive. It should be noted that the cumulative prediction accuracy may be inflated for cases where the first level code was determined with perfect accuracy using external knowledge. Consequently, it should be considered in conjunction with the values of the measure *A*(*k*) defined above. Doing so also ensures that we can track the variation in prediction accuracy as we traverse the ATC hierarchy. In the following this measure is denoted as *CA*(*k*). In the limit, *CA*(5) represents the overall prediction accuracy across all the levels of the ATC hierarchy.
- *Number of compounds correctly predicted to k* ^*th*^ *ATC level:* Reflects the percentage of compounds for which the entire ATC code was correctly predicted till level *k*. A compound is considered to have its ATC code correctly predicted to the *k* ^*th*^ level, if each level of that compound’s ATC code till level *k* inclusive has been correctly predicted. Hereafter, this measure is denoted as *N*(*k*).

Learning was performed using the training dataset and the efficacy of TL was assessed in terms of prediction accuracy for the second through fourth levels of the ATC code on the test set. As mentioned earlier, the prediction methods were initialized using supervised learning, predictions from SuperPred, and through first level ATC codes retrieved from ChEMBL. The last strategy led (by design) to the most accurate seeding of the learning process. For initialization using supervised learning, the first level ATC code predicted by the corresponding classifiers in the STP setting were used to seed the TL approach. The accuracy of initialization in terms of *A*(1) and *N*(1) values is provided in Table 5. In Tables 6, 7, and 8 we present the results for predicting the ATC codes using TL at the second, third, and fourth levels respectively.

**Table 5:** Accuracy of TL initialization at predicting the first level ATC code

*1* ^*nd*^ *level ATC Code Prediction initialization accuracy*			
*Initialization method*	*Supervised learning*	*SuperPred*	*ChEMBL (Perfect 1st level)*
Prediction method	*A(1), N(1)*	*A(1), N(1)*	*A(1), N(1)*
MLP	73.66%	77.95%	100%
Naive Bayes	47.67%	77.95%	100%
Random Forest	66.49%	77.95%	100%
SVM	72.22%	77.95%	100%

**Table 6:** Accuracy of TL for predicting the second level ATC code

*2* ^*nd*^ *level ATC Code Prediction using different initializations and comparison to STP*								
*Initialization method*	*Supervised learning*		*SuperPred*		*ChEMBL (Perfect 1st level)*		*STP*	
Prediction method	*A(2)*	*CA(2)* *(N(2))*	*A(2)*	*CA(2)* *(N(2))*	*A(2)*	*CA(2)* *(N(2))*	*A(2)*	*CA(2)* *(N(2))*
MLP	**69.18%**	**71.42%** **(64.34%)**	70.79%	74.37% (68.47%)	79.03%	89.52% (79.03%)	**69.89%**	**71.77%** **(67.56%)**
Naive Bayes	48.21%	47.94% (36.74%)	60.22%	69.09% (57.35%)	65.41%	82.71% (65.05%)	46.95%	47.31% (36.38%)
Random Forest	63.98%	65.23% (57.71%)	68.28%	73.12% (66.49%)	77.60%	88.80% (77.60%)	62.19%	64.34% (58.96%)
SVM	67.92%	69.89% (63.26)	**72.40%**	**75.18%** **(70.79%)**	**81.90%**	**90.95%** **(81.90%)**	68.46%	70.16% (64.52%)

The best STP results are included for comparison. The highest prediction accuracies obtained with each initialization strategy are highlighted in bold

**Table 7:** Accuracy of TL for predicting the third level ATC code

*3rd level ATC Code Prediction using different initializations and comparison to STP*								
*Initialization method*	*Supervised learning*		*SuperPred*		*ChEMBL (Perfect 1st level)*		*STP*	
Prediction method	*A(3)*	*CA(3)* *(N(3))*	*A(3)*	*CA(3)* *(N(3))*	*A(3)*	*CA(3)* *(N(3))*	*A(3)*	*CA(3)* *(N(3))*
MLP	63.62%	**68.82%** **(57.16%)**	63.26%	70.67% (58.24%)	72.40%	83.81% (66.67%)	**72.40%**	**73.42%** **(59.14%)**
Naive Bayes	56.99%	50.96% (31.00%)	66.49%	68.22% (46.59%)	69.00%	78.14% (52.51%)	57.53%	60.81% (30.47%)
Random Forest	57.35%	62.60% (48.03%)	60.93%	69.06% (54.84%)	69.00%	82.20% (62.37%)	65.23%	68.46% (50.18%)
SVM	**64.16%**	67.98% (56.99%)	**67.56%**	**72.64%** **(63.44%)**	**76.88%**	**86.26%** **(71.68%)**	64.52%	70.31% (58.60%)

The best STP results are included for comparison. The highest prediction accuracies obtained with each initialization strategy are highlighted in bold

**Table 8:** Accuracy of TL for predicting the fourth level ATC code

*4th level ATC Code Prediction using different initializations and comparison to STP*								
*Initialization method*	*Supervised learning*		*SuperPred*		*ChEMBL (Perfect 1st level)*		*STP*	
Prediction method	*A(4)*	*CA(4)* *(N(4))*	*A(4)*	*CA(4)* *(N(4))*	*A(4)*	*CA(4)* *(N(4))*	*A(4)*	*CA(4)* *(N(4))*
MLP	48.57%	**63.75%** **(37.81%)**	48.57%	65.14% (37.09%)	50.90%	75.58% (41.93%)	**51.61%**	67.97% (40.32)
Naive Bayes	36.92%	47.45% (19.35%)	45.70%	62.59% (29.03%)	46.77%	70.30% (32.80%)	38.35%	55.20% (22.76%)
Random Forest	40.68%	57.12% (29.93%)	47.49%	63.66% (33.87%)	50.36%	74.24% (38.53%)	39.43%	61.20% (28.49%)
SVM	**50.18%**	63.53% (40.68%)	**56.09%**	**68.50%** **(45.34%)**	**56.81%**	**78.90%** **(49.64%)**	41.22%	**65.37%** **(41.22%)**

The best STP results are included for comparison. The highest prediction accuracies obtained with each initialization strategy are highlighted in bold

In analyzing these results, it is clear that for TL correctly predicting the first level of the ATC code is important. Furthermore, excluding MLP, all prediction methods when incorporated in TL with either SuperPred-based initialization or initialization using ChEMBL, performed better than under STP. These improvements translated into an increase in the number of compounds correctly predicted, as detailed in Table 9. The greatest improvement occurred for the Naïve Bayes method while SVM consistently outperformed other predictors. While TL improved the prediction accuracy of the MLP at the second ATC level, it resulted in drop in accuracy at the third level and fourth level when initialized with SuperPred. The decline in the performance of MLP in the TL framework may indicate the need for larger training sets at each level to optimally tune the relevant MLP parameters. The reader may note however, that the performance of the most accurate predictor (SVM) in the TL framework outperformed, by far, the highest prediction accuracy achieved with MLP under STP.

**Table 9:** Comparison of the predictive performance of TL and STP

*Comparison of CA(k) using STP and TL at the second, third, and fourth ATC levels*									
	*STP N(2)*	*N(2)* *SuperPred* (+/−)	*N(2)* *ChEMBL* (+/−)	*STP CA(3)*	*N(3)* *SuperPred* (+/−)	*N(3)* *ChEMBL* (+/−)	*STP CA(4)*	*N(4)* *SuperPred* (+/−)	*N(4)* *ChEMBL* (+/−)
MLP	377	382 (+5)	383 (+6)	330	325 (−5)	372 (+42)	225	207 (−18)	234 (+9)
Naive Bayes	203	320 (+117)	363 (+160)	170	260 (+90)	293 (+123)	127	162 (+35)	183 (+56)
Random Forest	329	371 (+42)	431 (+102)	280	306 (+26)	348 (+68)	159	189 (+30)	215 (+56)
SVM	360	**395** **(+35)**	**457** **(+97)**	327	**354** **(+27)**	**400** **(+73)**	230	**253** **(+23)**	**277** **(+47)**

This table compares the number of compounds underlying *N(k)* for each classifier in predicting the second through fourth level of the ATC code using TL and STP. Only the TL initializations using SuperPred and ChEMBL and shown. The change (+/−) is noted below the numbers underlying *N(k)* for each level. The best results are highlighted for each initialization strategy

Finally, if the prediction accuracy of the TL process is analyzed by only considering the cases where the supervised learning-based initialization led to the correct first level ATC code, then the advantage of TL over STP becomes stark. These results are summarized in Table 10 and show that TL matched or outperformed STP on all classifiers at all levels except for MLP at level four, where the TL prediction accuracy marginally lagged behind that of STP.

**Table 10:** Comparison of STP and TL prediction accuracy when TL was initialized with correct ATC codes

*Comparison of N(k) of STP and TL when compared on Supervised learning initialization dataset*						
	STP *N(*2*)*	TL *N(*2*)* (+/−)	STP *N(*3*)*	TL *N(*3*)* (+/−)	STP *N(*4*)*	TL *N(*4*)* (+/−)
MLP	85.40%	87.35% (1.95%)	77.62%	77.62% (0.00%)	52.07%	51.34% (−.73%)
Naive Bayes	57.69%	78.85% (21.15%)	48.85%	66.54% (17.69%)	31.92%	41.54% (9.62%)
Random Forest	81.03%	87.26% (6.23%)	70.46%	72.63% (2.17%)	39.84%	45.26% (5.42%)
SVM	83.29%	88.03% (4.74%)	76.06%	79.30% (3.24%)	53.87%	56.61% (2.74%)

This table compares *N*(*k*) values for STP and TL initialized with supervised learning at the second through fourth levels of the ATC code

### Comparison of TL with extant ATC code prediction methods

To compare the accuracy of TL with Chen et al. [5], we note that the highest accuracy at the first level obtained by us was 73.66% using the MLP classifier on the test set (Table 3). In comparison, Chen *et al*, reported an accuracy of 75.9% on their training set and an accuracy of 66.36% on their test set. Further, when using structural similarity as the only feature, the accuracy of the proposed method was 53.05% at the first ATC level, which is also substantially higher than the 40% accuracy reported in [5]. Finally, Chen et al. do not report predictions of ATC codes beyond the first level. Consequently a direct comparison with TL is infeasible.

A comparison of the proposed approach with NetPredATC [11], which reported prediction accuracy values ranging between 74 and 76.5% was complicated due to the fact that at the time of writing this paper, only a preliminary implementation of NetPredATC was available. This implementation was suitable for limited testing which prevented direct comparisons. It should also be noted here that the accuracy measure in [11] was defined as the ratio of the sum of true positive and true negative predictions to the sum of true positive, true negative, false positive, and false negative predictions. As such, this definition of accuracy is distinct from the measures *A*(*k*), *CA*(*k*) and *N*(*k*) used by us.

In order to directly compare our method to SuperPred, we identified 204 compounds in our test set that were not present in the database of the SuperPred webserver. In the following, we term this as the reduced test set. The prediction performance for both methods on this dataset is presented in Tables 11, 12, 13 and 14. TL with ChEBML initialization was found to outperform SuperPred at all levels. For the supervised learning-based initialization, TL had higher *N*(*k*) values for the for the first second, and third levels than SuperPred. However, at the fourth level, the *N*(*k*) accuracy of SuperPred exceeded that for TL.

**Table 11:** Comparison of the prediction accuracy of TL and SuperPred for the first level ATC codes

*Accuracy of 1* ^*nd*^ *level ATC Code Prediction*		
*Initialization method*	*Supervised learning*	*ChEMBL (Perfect 1st level)*
Prediction method	*A(1)* *(N(1))*	*A(1)*
MLP	54.41% (54.41%)	100%
Naive Bayes	33.82% (33.82%)	100%
Random Forest	52.45% (52.45%)	100%
SVM	**55.39%** **(55.39%)**	100%
SuperPred Prediction Results	42.16% (42.16%)	---

Results are for the reduced test set. The highest numerical accuracies are highlighted in bold

**Table 12:** Comparison of the prediction accuracy of TL and SuperPred for the second level ATC codes

*2* ^*nd*^ *level ATC Code Prediction using different initializations and comparison to STP*						
*Initialization method*	*Supervised learning*		*ChEMBL (Perfect 1st level)*		*SuperPred Prediction Results*	
Prediction method	*A(2)*	*CA(2)* *(N(2))*	*A(2)*	*CA(2)* *(N(2))*	*A(2)*	*CA(2)* *(N(2))*
MLP	50.00%	52.21% (43.63%)	65.20%	82.60% (53.39%)	44.61%	43.38% (39.22%)
Naive Bayes	41.67%	37.35% (25.00%)	51.47%	75.74% (51.47%)		
Random Forest	49.51%	50.98% (42.16%)	64.71%	82.35% (65.20%)		
SVM	**52.45%**	**53.92%** **(45.10%)**	**67.16%**	**83.58%** **(67.65%)**		

Results are for the reduced test set. The highest accuracy obtained with TL is highlighted in bold

**Table 13:** Comparison of the prediction accuracy of TL and SuperPred for the third level ATC codes

*3* ^*rd*^ *level ATC Code Prediction using different initializations and comparison to STP*						
*Initialization method*	*Supervised learning*		*ChEMBL (Perfect 1st level)*		*SuperPred Prediction Results*	
Prediction method	*A(3)*	*CA(3)* *(N(3))*	*A(3)*	*CA(3)* *(N(3))*	*A(3)*	*CA(3)* *(N(3))*
MLP	**46.08%**	**50.16%** **(46.07%)**	64.71%	62.95% (52.45%)	48.53%	45.10% (38.73%)
Naive Bayes	60.29%	45.26% (21.57%)	**66.67%**	72.71% (40.69%)		
Random Forest	41.18%	47.71% (32.35%)	61.27%	75.33% (49.51%)		
SVM	42.65%	50.98% (37.75%)	66.18%	**77.78%** **(54.41%)**		

Results are from the reduced test set. The highest accuracy obtained with TL is highlighted in bold

**Table 14:** Comparison of the prediction accuracy of TL and SuperPred for the fourth level ATC codes

*4th level ATC Code Prediction using different initializations and comparison to STP*						
*Initialization method*	*Supervised learning*		*ChEMBL (Perfect 1st level)*		*SuperPred Prediction Results*	
Prediction method	*A(4)*	*CA(4)* *(N(4))*	*A(4)*	*CA(4)* *(N(4))*	*A(4)*	*CA(4)* *(N(4))*
MLP	**39.71%**	47.55% (22.55%)	47.55%	69.36% (33.82%)	56.86%	48.04% (38.24%)
Naive Bayes	35.78%	42.89% (15.20%)	45.59%	65.93% (27.94%)		
Random Forest	35.29%	44.61% (21.57%)	**49.51%**	68.87% (33.82%)		
SVM	38.24%	**47.79%** **(27.94%)**	49.02%	**70.59%** **(38.73%)**		

Results are from the reduced test set. The highest accuracy obtained with TL is highlighted in bold

## Conclusion

The ability to predict *in silico*, the ATC code of an arbitrary compound with high accuracy can help in library construction for lead identification, and assist in drug repositioning. Taken together, these advantages can reduce late-stage drug failure and significantly impact the cost of drug discovery.

In this paper, we have presented tiered learning–a methodology for predicting the ATC code of a compound. Experimental studies conducted by us indicate the promise of the proposed approach: most of the learning algorithms that employed the proposed architecture experienced significant improvement in prediction performance. When employed with high-quality initializations, TL was found to either improve upon or be comparable to other methods at the state-of-the-art with whom direct comparisons were possible. Separately, in an experiment that involved randomly labeling the 558 compounds in the test set, the prediction accuracy dropped below 13% for the first level ATC code using STP, underlining the validity of the learning formulation.

The high prediction accuracy possible with the proposed approach makes TL a potentially viable technology for low-cost *in-silico* prediction of ATC codes of compounds prior to employing expensive and time-consuming biochemical assays. While computational prediction of drug properties such as bioavailability and ADME-PK is often employed in the drug-discovery pipeline, the high prediction accuracy obtained through the use of the proposed learning architecture indicates that computationally predicted information-rich drug descriptors, such as ATC codes, can also be employed, especially in library construction for lead identification.
